# An international Delphi consensus process to determine a common data element and core outcome set for frailty: FOCUS (The Frailty Outcomes Consensus Project)

**DOI:** 10.1186/s12877-022-02993-w

**Published:** 2022-04-05

**Authors:** Jeanette C. Prorok, Paula R. Williamson, Beverley Shea, Darryl Rolfson, Leocadio Rodriguez Mañas, Matteo Cesari, Perry Kim, John Muscedere

**Affiliations:** 1 Canadian Frailty Network, Kingston, ON Canada; 2 University of Liverpool, Liverpool, UK; 3 University of Ottawa, Ottawa, Canada; 4 University of Alberta, Edmonton, Canada; 5 Hospital Universitario de Getafe ES, Madrid, Spain; 6 University of Milan, Milan, Italy; 7 Queen’s University, 99 University Avenue, Kingston, ON K7L 3N6 Canada; 8 Kingston Health Sciences Center, 76 Stuart Street, Ontario K7L 2V7 Kingston, Canada

**Keywords:** Frailty, Common data elements, Core outcome set, Delphi, COMET, OMERACT

## Abstract

**Background:**

Despite increased recognition of frailty and its importance, high quality evidence to guide decision-making is lacking. There has been variation in reported data elements and outcomes which makes it challenging to interpret results across studies as well as to generalize research findings. The creation of a frailty core set, consisting of a minimum set of data elements and outcomes to be measured in all frailty studies, would allow for findings from research and translational studies to be collectively analyzed to better inform care and decision-making. To achieve this, the Frailty Outcomes Consensus Project was developed to reach consensus from the international frailty community on a set of common data elements and core outcomes for frailty.

**Methods:**

An international steering committee developed the methodology and the consensus process to be followed. The committee formulated the initial list of data elements and outcomes. Participants from across the world were invited to take part in the Delphi consensus process. The Delphi consisted of three rounds. Following review of data after three rounds, a final ranking round of data elements and outcomes was conducted. A required retention rate of 80% between rounds was set a priori.

**Results:**

One hundred and eighty-four panelists from 25 different countries participated in the first round of the Delphi consensus process. This included researchers, clinicians, administrators, older adults, and caregivers. The retention rate between rounds was achieved. Data elements and outcomes forming primary and secondary core sets were identified, within the domains of participant characteristics, physical performance, physical function, physical health, cognition and mental health, socioenvironmental circumstances, frailty measures, and other.

**Conclusion:**

It is anticipated that implementation and uptake of the frailty core set will enable studies to be collectively analyzed to better inform care for persons living with frailty and ultimately improve their outcomes. Future work will focus on identification of measurement tools to be used in the application of the frailty core set.

**Supplementary Information:**

The online version contains supplementary material available at 10.1186/s12877-022-02993-w.

## Background

As the proportion of older adults in the global population rises, there has been increased focus on frailty [1]. The World Health Organization defines frailty as “a clinically recognizable state in which the ability of older people to cope with every day or acute stressors is compromised by an increased vulnerability brought by age-associated declines in physiological reserve and function across multiple organ systems” [2]. In spite of increased recognition of frailty and its importance, high quality evidence to guide decision-making is lacking. This is due to a number of reasons, including the exclusion of older adults living with frailty from research studies, lack of consideration of the differential impact of frailty within research studies, a poor understanding of frailty and its measurement, and varying performance of the tools used to assess frailty in different clinical and social settings [3–6]. Additionally, there has been variation in reported data elements and outcomes which makes it challenging to interpret results across studies as well as to generalize research findings. The creation of a frailty core set, consisting of a minimum set of data elements and outcomes to be measured in all frailty studies, would allow for findings from research and translational studies to be collectively analyzed to better inform care and decision-making. Core sets of data elements and outcome measures have been developed for numerous other diseases and conditions [7–11], however no such set currently exists for frailty.

To address this, in 2018, an international group of experts met to discuss the path forward for the development and use of common data elements and core outcomes in future frailty studies. A summary of the meeting’s discussions, including analysis of the need for a frailty core set, was published [12]. It was determined that a transparent, international consensus initiative be undertaken to determine a core frailty set. In late 2019, the Frailty Outcomes Consensus (FOCUS) Project was launched. Using a Delphi methodology, FOCUS aimed to bring the international frailty community to consensus on a set of common data elements and core outcomes for frailty, thereby meeting a pressing need in the frailty research community. The results of the Delphi consensus process as well as future directions for the implementation and adoption of the frailty core set are presented.

## Methods

### Generation of data elements and outcomes list

Following the Outcome measures in Rheumatology (OMERACT—www.omeract.org) and Core Outcome Measures in Effectiveness Trials (COMET –www.comet-initiative.org) [13] methodologies, the first step in the Delphi process was the generation of an initial list of data elements and outcomes for the voting consensus process. Data elements were defined as study population descriptors/characteristics. A review of the literature was conducted to identify common data elements and outcomes reported in frailty-related randomized controlled trials (unpublished data). Additionally, an online survey was broadly distributed for input on data elements and outcomes to be included in the initial list. This allowed for broad consultation with researchers and clinicians working in the field of frailty. In order to ensure that data elements and outcomes of importance to older persons living with frailty and their caregivers were included, an in-person mixed-methods survey was administered to persons living with frailty and their caregivers (paid and unpaid caregivers). The survey was administered both in the community setting and long term care setting. In this manner, the research literature, survey input from researchers and clinicians, as well as input from persons living with frailty and their caregivers informed the development of a comprehensive list of data elements and outcomes for the Delphi consensus process. Instrument selection for determining these measures was not part of this process but will be the topic of future work.

### Formation of steering committee and identification of Delphi panel members

A steering committee was formed to guide the Delphi process and met approximately quarterly. The steering committee consisted of international experts in frailty [DR, LRM, MC, JM] and Delphi methodology [PW, BS]. The steering committee reviewed and confirmed the list of data elements and outcomes for inclusion in the Delphi consensus process. Additionally, the steering committee informed key decisions in the process, such as defining the criteria for consensus, the number of rounds, and provided ongoing monitoring of aggregate level data to determine when consensus was reached at the conclusion of the Delphi process.

A Delphi panel was also convened. The Delphi panelists were the individuals who voted and participated in the consensus process. As the objective of the FOCUS project was to facilitate international consensus on data elements and outcomes for frailty, a broad geographic representation of participants was sought. We aimed to include panelists from numerous sectors, including research, clinical, policy, industry, and regulatory bodies. We also sought to include persons living with frailty and their caregivers as Delphi panelists, however participants must have been able to read and write in English and use a computer to participate. Panelists were identified through CFN’s broad network of members, as well as through targeted searches to identify potential panelists from regions not yet well represented in the participant sample.

### Delphi consensus process

**Fig. 1:**
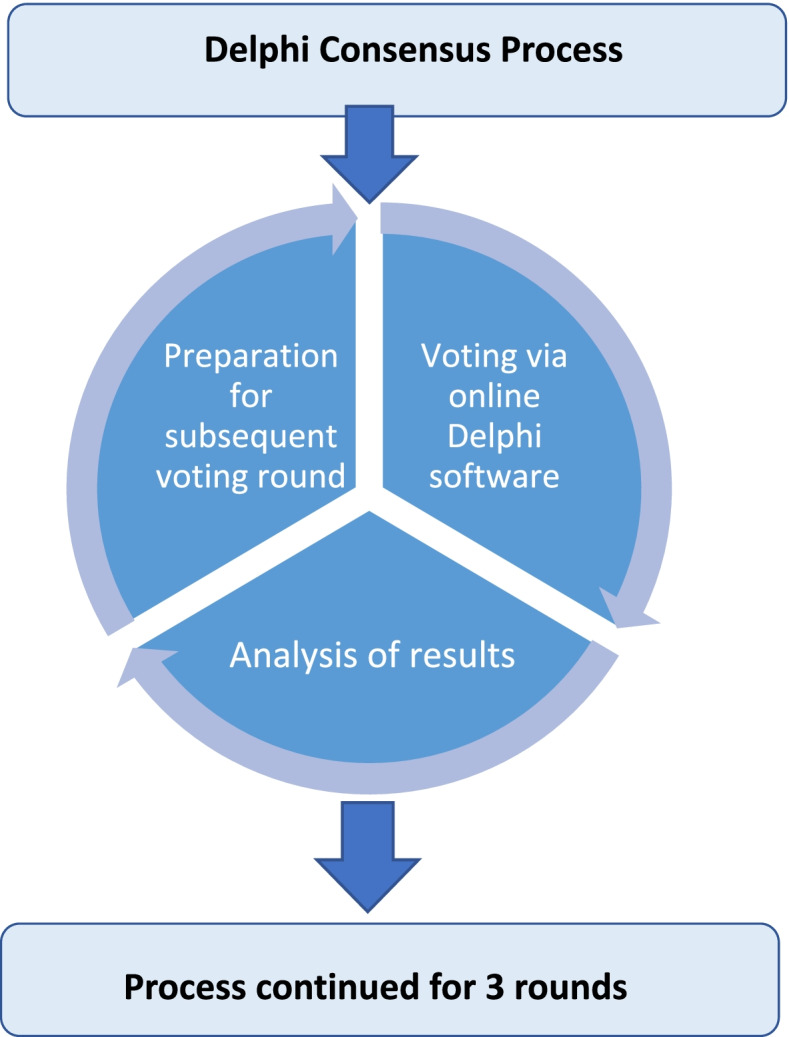
FOCUS Delphi consensus process

The FOCUS project is registered in the COMET (Core Outcome Measures in Effectiveness Trials) database [https://www.comet-initiative.org/Studies/Details/1364 ethics] and received approval through the Health Sciences Research Ethics Board (HSREB) at Queen’s University (CCM-020–19). All study protocols were carried out in accordance with Queen’s HSREB guidelines and Tri-Council Policy ethics regulations. Informed consent was obtained from all panelists prior to their participation. Panelists were invited via email to take part in the Delphi consensus process. A summary of the Delpih process is depicted in Fig. 1. Due to its global reach spanning most time zones, the Delphi was conducted online via a survey administered through Delphi consensus software (DelphiManager, www.comet-initiative.org/delphimanager). Upon providing consent to study participation and completion of registration, a webinar was made available to panelists. The webinar provided an all-important introduction to the study, serving to orientate panelists to the goals of the consensus process and to familiarize panelists with the project. The webinar also provided logistical information regarding survey administration and completion.

Panelists then proceeded to complete the first round of the Delphi process. Data elements and outcomes were organized into domains. A definition or example was provided for each data element or outcome to participants. Panelists scored each data element or outcome on a scale from one to nine. A score between one to three indicated that the panelist felt that data element or outcome was not important; four to six indicated important but not critical; and a score between seven and nine indicated that the data element or outcome was critical to include in the frailty set. Panelists could also provide feedback if they wished on any data element or outcome, though limited feedback was received. In Round 1, panelists were also given the opportunity to share any additional data elements or outcomes which they felt should be included. Following completion of the first round, the data were analyzed and the consensus criteria predetermined by the steering committee were applied (Table 1). These criteria had been developed by the OMERACT group (Outcome Measures in Rheumatology) and adapted for the FOCUS project [14]. Data were analyzed separately by stakeholder group (older adults; caregivers; researchers/clinicians/administrators). This was to ensure that data elements and outcomes of importance to older adults and caregivers were not missed due to being outnumbered by the other stakeholder group. Researchers, clinicians, and administrators were grouped together as one stakeholder group, as individuals may often hold more than one of these roles (ex. a clinician-researcher).

**Table 1:** Consensus criteria identified for FOCUS Delphi process ( adapted from OMERACT) [13]

Consensus Criteria	
1. Consensus that a data element/outcome is important for a core domain set: ≥ 80% of participants in all groups scored the item as "critically important to include in a core set" (score 7 to 9) and < = 10% score as 1–3; these items are acknowledged in subsequent rounds as having met criteria for importance to a core set, and held for final round discussion	2. Consensus that a data element/outcome will NOT be included: ≥ 50% of participants in all groups scored the item as of "limited importance" (score 1 to 6); these items are dropped from Delphi and are not to be part of core set
3. Dissensus but important to one group: 80% + participants in one of our groups score items as critically important for a core set (score 7 to 9); data element/outcome continues on to next round as having no consensus yet; if data element/outcome does not reach consensus level at end of Delphi, but still important to one group, it will be held for final round discussion	4. No consensus: All other results; data element/outcome continues to next round as having no consensus yet. If data element/outcome does not achieve consensus by last round, and no groups have supported it ≥ 80%, then data element/outcome is not endorsed for core set

Data elements and outcomes requiring further voting then underwent scoring once again in round two. It was anticipated that there would be attrition between rounds. A priori, the steering committee had set a minimum required retention rate of 80% between rounds. As with the first round, panelists scored the data element or outcome on a scale of one to nine. Panelists were reminded of their scores from the previous round. Data were analyzed in the same manner as in the first round and consensus criteria were applied. As consensus had not yet been reached after the second round, panelists completed the same process again for a third round of voting. Following three rounds of voting, the steering committee met to review the results. There was concern that the number of data elements and outcomes which had met the criteria for consensus was high for a frailty core set and that it would be unreasonable to expect users of the core set to measure all of the data elements and outcomes identified. Consequently, the steering committee elected to conduct a fourth round in which panelists would rank their top two data elements or outcomes within each domain. This decision was consistent with accepted methodology recommended to prioritize data elements and outcomes when a large number remain following the Delphi consensus process [13]. The data element or outcome in each domain which received the most rankings as the top data element or outcome formed part of the primary core set, while the data element or outcome in each domain receiving the most rankings as the second most important data element or outcomes formed part of the secondary core set. Following the ranking round, a panel of key stakeholders from around the world was convened to review the final results, in addition to the steering committee. While this was initially planned to be an in-person meeting, due to the ongoing pandemic restrictions the stakeholder meeting was conducted virtually. Stakeholders provided feedback on the final frailty core set.

## Results

### Panelist characteristics

**Fig. 2:**
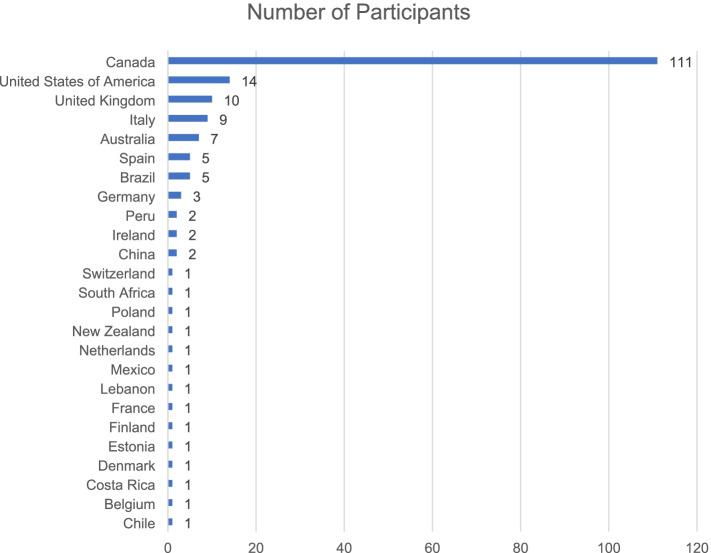
Number of panelists by country

**Fig. 3:**
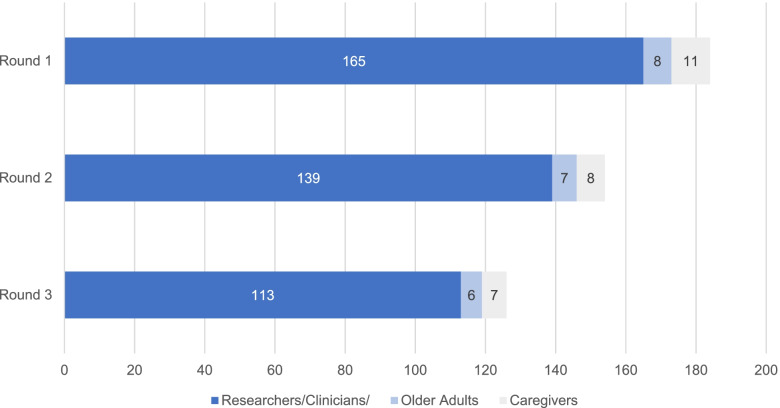
Number of panelists by stakeholder group in each Delphi round. * 1 caregiver passed away during the course of the study; therefore the caregiver denominator was 10 for round 2

One hundred and eighty-four panelists from 25 different countries participated in the first round of the Delphi consensus process, with 60% of panelists identifying themselves as participating from Canada (Fig. 2). The number of panelists per stakeholder group in each round of the Delphi is presented in Fig. 3. The a priori set criteria of 80% retention between rounds was achieved.

Table 2 presents the available demographic characteristics of panelists. It should be noted that amongst the researcher/clinician/administrator group, though panelists had identified themselves as such for the purpose of the Delphi, 54 panelists in this stakeholder group also indicated that they are caregivers of a family member or friend living with frailty.

**Table 2:** Demographic characteristics of panelists

Stakeholder Group	Mean Age in years (Range)	Gender^a^ (Male:Female)
Researchers/Clinicians/Administrators	50.1 (27–76)	61:104
Older Adults	74.1 (70–83)	4:4
Caregivers	61.0 (41–85)	1:10

^a^Panelists did not identify as any gender other than male/female, though options were available for selection

### Delphi results

Tables 3 and 4 present the data elements, meeting consensus criteria across all three stakeholder groups after three rounds of the Delphi consensus process (organized by domain) and the data elements forming the core set, respectively. Similarly, Tables 5 and 6 present the outcomes meeting consensus criteria across all three stakeholder groups and the core set of outcomes, respectively. Panelists were asked to rate items from one to nine as both a data element and as an outcome. As the tables demonstrate, in some cases panelists felt an item was critical to include as a data element but not an outcome or vice versa, and in some instances as both. Overall, 28 data elements across 8 domains met the consensus criteria in all three stakeholder groups. Seventeen outcomes across 7 domains met the consensus criteria in all three stakeholder groups. Similar across both data elements and outcomes, the most items meeting consensus criteria were found in the Physical Function Domain, with many of these items overlapping in both the data element and outcome groups.

**Table 3:** Data elements fulfilling consensus criteria in all three stakeholder groups after three rounds

Domain	Data Element
Participant Characteristics	Age
	Medications
Physical Performance	Balance
	Mobility
Physical Function	Activities of daily living
	Disability
	Falls
	Function – lower body
	Function – upper body
	Instrumental activities of daily living
	Overall function
	Physical activity
Physical Health	Comorbidities
	Nutritional status
	Sensory impairment
	Visual impairment
Cognition and Mental Health	Anxiety
	Cognitive impairment
	Delirium
	Depression
	Psychosocial function
Socioenvironmental Circumstances	Formal care services
	Informal care and support
	Physical isolation
	Social engagement
Approach to Frailty Measurement	Cumulative deficit
	Multi-dimensional
Other	Quality of life

**Table 4:** Primary and secondary data elements identified for frailty core set following ranking process

Domain	Data Element	
	**Primary**	**Secondary**
Participant Characteristics	Age	Medications
Physical Performance	Mobility	Balance
Physical Function	Activities of daily living	Overall function
Physical Health	Comorbidities	Nutritional status
Cognition and Mental Health	Cognitive impairment	Psychosocial function
Socio-environmental Circumstances	Informal care and support	Formal care services
		Social engagement
Frailty Measures	Cumulative deficit	Multi-dimensional
Other	Quality of life

**Table 5:** Outcomes fulfilling consensus criteria in all three stakeholder groups after three rounds

Domain	Outcome
Physical Performance	Balance
Physical Function	Activities of daily living
	Disability
	Falls
	Instrumental activities of daily living
	Overall function
	Physical activity
Cognition and Mental Health	Cognitive impairment
	Depression
Socioenvironmental Circumstances	Informal care and support
	Physical isolation
Approach to Frailty Measurement	Cumulative deficit
	Multi-dimensional
	Physical performance
Other	Burden of intervention received
	Quality of life
	Caregiver characteristics

**Table 6:** Primary and secondary outcomes identified for frailty core set following ranking process

Domain	Outcome	
	**Primary**	**Secondary**
Physical Performance	Balance	
Physical Function	Overall function	Activities of daily living
Cognition and Mental Health	Cognitive impairment	Depression
Socio-environmental circumstances	Informal care and support	Physical isolation
Frailty Measures	Cumulative deficit	Multi-dimensional
Other	Quality of life	Caregiver characteristics

Given the measurement burden that would be likely with a set of data elements and outcomes of this size, it was determined that a final ranking round would be conducted. Panelists were asked to rank their top two data elements and outcomes within any domain which had two or more data elements or outcomes meeting consensus criteria across the three stakeholder groups. Sixty-four researchers/clinicians/administrators, four caregivers, and two older adults agreed to participate in the additional ranking round. This resulted in a final core set of 14 primary data elements and outcomes, and 10 secondary primary data elements and outcomes. While the measurement of 24 data elements and outcomes may still seem significant, it should be noted that there is overlap between the identified data elements and outcomes. For example, balance has been identified as both as data element and an outcome in the core set.

A stakeholder panel of twenty individuals from around the world met virtually to review the results of the ranking round. Panel discussions helped to confirm the results of the ranking round. Additionally, the panel provided insight regarding facilitating the uptake and implementation of the frailty core set globally, including potential challenges. These are presented as part of the discussion in the following section.

## Discussion

The Delphi consensus process carried out in this study has resulted in a frailty core set of data elements and outcomes for use in future frailty research. It has identified 14 primary data elements and outcomes and 10 secondary data elements and outcomes. These data elements and outcomes fall within the domains of participant characteristics, physical performance, physical function, physical health, cognitive and mental health, socio-environmental circumstances, and frailty measures. It is anticipated that frailty status of participants could be derived from the measurement of data elements and outcomes across these domains. As stated, several items were identified as both a data element and an outcome in the core set. These items included: balance, activities of daily living, overall function, cognitive impairment, informal care and support, quality of life, as well as cumulative deficit and multi-dimensional approaches to frailty measurement. The identification of these items as critical to include as both data elements and outcomes may provide an indication of how important these specific data elements and outcomes were to panelists.

This Delphi study contributes to an area of the frailty research literature that to date has not been addressed. To our knowledge, this is the first frailty core set developed. It was developed through the participation of not only researchers, clinicians, and administrators, but also older adults and caregivers ensuring that the data elements and outcomes included in the final set were found to be important by all three stakeholder groups. This is of particular importance, as in the past outcome selection for trials and research studies was typically done by researchers and/or clinicians, without the input of patients or other stakeholders [15]. However, increasingly there is recognition that data elements and outcomes identified for use in research studies must be relevant to a wider range of stakeholders, including patients and caregivers [16–18]. In fact, the inclusion of patients in the development of core outcome sets has been identified as part of the Core Outcome Set – STAndards for Development: The COS-STAD recommendations [19].

The inclusion of a global stakeholder panel to review the resulting frailty core set resulted in the identification of several key considerations, particularly with regard to the implementation of the set. The first consideration is to be cognizant of the context in which the set is being applied. The stakeholder panel represented a variety of settings and expressed that some data elements or outcomes may be more relevant in their respective settings than others. Frailty research studies may take place in a number of different settings, ranging from clinical to non-clinical, including acute care, long term care, and community settings. Although we urge the collection of the primary data elements, we recognize that there may be setting and resource limitations for users selecting between the primary and secondary data elements and outcomes for use in their studies. Only if required by unmodifiable circumstances, users of the set may wish to select the data elements or outcomes which are most applicable.

Similarly, the stakeholder panel pointed out that there may be country or region specific differences and preferences when utilizing the frailty core set in research studies around the world. This was very apparent in discussions regarding the approach to frailty measurement. The consensus process results showed a cumulative deficit approach to be preferred, followed by multi-dimensional measures of frailty. A cumulative deficit approach was defined as a quotient of deficits present from a predetermined list of at least 30 available in the health record (ex. Frailty Index), while a multi-dimensional approach to measurement was a clinician-oriented set of items that comprise multiple frailty domains (ex. Edmonton Frail Scale). As stakeholders pointed out, the cumulative deficit approach may have been preferred due to the predominantly Canadian sample. In the European context, these stakeholders did not see this approach as preferable. This feedback indicates that there may be challenges in uptake of this data element/outcome in these contexts. It also offers a signal that perhaps further research and analysis is needed regarding preferred approaches to frailty measurement around the world. Stakeholder panelists encouraged follow-up work in this area.

The stakeholder panel also discussed measurement of the identified data elements and outcomes in the frailty core set. The stakeholder panel indicated that it will be important to consider the responsiveness to change of measurement tools used for assessment of data elements and outcomes in the frailty core set. Future directions of the FOCUS project include garnering consensus on measurement tools or instruments for the implementation of the frailty core set. This will be critical to ensure that not only are frailty studies measuring the same data elements and outcomes to facilitate cross-study comparisons, but also that these data elements and outcomes are being measured in a consistent manner. Selection of measurement tools was beyond the scope of work of this Delphi study, as measurement tool selection will require its own consensus-based processes [20].

Though more than half of the sample of participants was Canadian, the Delphi process was strengthened by the inclusion of perspectives from around the world and the global frailty community. It should be noted however, that the representativeness of the sample is limited by the inclusion of English-speaking participants with access to a device/internet. Further work with a more international audience could serve to enrich and validate the results of this Delphi process. Additionally, the process would have benefited from the inclusion of an older adult and/or caregiver on the steering committee to help guide the process with their input. The consensus process followed previously established methodology, developed by recognized core outcome set development groups and was registered in the COMET database.

## Conclusions

The FOCUS study aimed to identify a core set of data elements and outcomes to facilitate the measurement of frailty-related data elements and outcomes across research studies. It is anticipated that implementation and uptake of the frailty core set will enable studies to be collectively analyzed to better inform care for persons living with frailty and ultimately improve their outcomes. In spite of its limitations, the FOCUS study provided the necessary first step toward consensus in the global frailty community. Future work will focus on identification of measurement tools to be used in the application of the frailty core set.

## Supplementary Information

**Additional file 1.**

## Data Availability

The data supporting the conclusions of this article is included within the article and its additional file.
