# Supplementary material for: An international Delphi consensus process to determine a common data element and core outcome set for frailty: FOCUS (The Frailty Outcomes Consensus Project)
Source: BMC Geriatr. 2022 Apr 5;22:284. doi: 10.1186/s12877-022-02993-w (PMC8985339; doi:10.1186/s12877-022-02993-w)
Supplement: Supplementary file 1 — Additional file 1. [file 12877_2022_2993_MOESM1_ESM.docx]

**Additional file 1: FOCUS Ranking Round Summary of Results**

| **Data Elements** | | | | |
| --- | --- | --- | --- | --- |
| **Category** | **HCP Rank 1 (n=64)** | **CG/OA Rank 1 (n=6)** | **HCP Rank 2 (n=64)** | **CG/OA Rank 2 (n=6)** |
| *Participant Characteristics* | Age (66%) | Age (67%) | Medications (34%) | Medications (72%) |
| *Physical Performance* | Mobility (73%) | Mobility (50%) | Balance (73%) | Balance (50%) |
| *Physical Function* | ADLs (42%) | ADLs (67%) | ADLs (50%) | Overall Function (67%) |
| *Physical Health* | Comorbidities (75%) | Nutritional Status (50%) | Nutritional Status (45%) | Nutritional Status (50%) |
| *Cognition and Mental Health* | Cognitive Impairment (78%) | Cognitive Impairment (67%) | Psychosocial Function (34%) | Psychosocial Function (33%) |
| *Socioenvironmental Circumstances* | Informal care support (28%) | Informal care and support (67%) | Social engagement (28%) | Formal care services (50%)/Social engagement (50%) |

| **Outcomes** | | | | |
| --- | --- | --- | --- | --- |
| **Category** | **HCP Rank 1 (n=64)** | **CG/OA Rank 1 (n=6)** | **HCP Rank 2 (n=64)** | **CG/OA Rank 2 (n=6)** |
| *Physical Function* | Overall function (38%) | ADLs (50%) | IADLs (25%) | ADLs (50%) |
| *Cognition and Mental Health* | Cognitive Impairment (84%) | Cognitive impairment (100%) | Depression (16%) | Depression (100%) |
| *Socioenvironmental Circumstances* | Informal care and support (64%) | Informal care and support (100%) | Physical Isolation (36%) | Physical Isolation (83%) |
| *Frailty Measures* | Cumulative deficit (44%) | Cumulative deficit (67%) | Multidimensional (44%) | Multidimensional (50%) |
| *Other* | Quality of life (81%) | Quality of life (100%) | Caregiver characteristics (44%) | Caregiver characteristics (50%) |
